# A significant increase in expression of FOXP3 and IL-17 genes in patients with allergic rhinitis underwent accelerated rush immunotherapy

**DOI:** 10.22038/ijbms.2019.32979.7878

**Published:** 2019-09

**Authors:** Amirabbas Salmani, Mojgan Mohammadi, Reza Farid Hosseini, Jalil Tavakol Afshari, Ali Fouladvand, Sajad Dehnavi, Maryam Khoshkhooi, Farahzad Jabbari Azad

**Affiliations:** 1 Department of Immunology, Faculty of Medicine, Mashhad University of Medical Sciences, Mashhad, Iran; 2 Allergy Research Center, Mashhad University of Medical Sciences, Mashhad, Iran; 3 Immunology Research Center, Mashhad University of Medical Sciences, Mashhad, Iran

**Keywords:** Accelerated rush – immunotherapy, Allergic rhinitis, FOXP3, Gene expression, IL-17

## Abstract

**Objective(s):**

Allergic rhinitis (AR) is a common hypersensitivity disease worldwide. Immunotherapy has been performed as the best treatment for years. This study aimed to study the gene expression pattern of immune system cells following an accelerated rush immunotherapy protocol (ARIT) in patients with AR.

**Materials and Methods:**

Fifteen patients with AR (15–55 years old) resident in Mashhad, Iran, with positive prick test to regional aeroallergens (weed mix, grass mix, tree mix, and Salsola) enrolled in this study. All patients were treated for three months with 3-day ARIT protocol between July 2015 and August 2016. Clinical symptoms and quality of life were recorded by two questioners. The expression levels of FOXP3, TGF-β, IL-10, IL-17, IL-4, and IFN-γ genes in patient’s peripheral blood mononuclear cells were evaluated by SYBR Green real-time RT-PCR technique.

**Results:**

The severity of disease and quality of life showed significant improvement following ARIT (*P*-value<0.05). Gene expression of IFN-γ and IL-10 was increased whereas TGF-β and IL-4 down-regulated, following ARIT, but these changes were not significant. However, gene expression of FOXP3 and IL-17 was significantly increased after intervention when compared with the baseline (*P*-value< 0.002).

**Conclusion:**

Significant up-regulation of FOXP3 and IL-17 genes, additionally, a significant improvement in the clinical signs following ARIT might be related to increases in HLA-DR- and FOXP3+ Treg population at the initiation phase of ARIT. Employing the flow cytometry technique to study the phenotype of these cells is suggested for future studies.

## Introduction

Allergic rhinitis (AR) is a common IgE-mediated hypersensitivity disease around the world, from which approximately 10 to 20% of world population suffer, and its prevalence is increasing, especially in industrial societies (1). The cause of AR is chronic inflammation in the upper respiratory tract due to inappropriate immune system response to innocent environmental allergens (2, 3). AR has four cardinal symptoms, including watery rhinorrhea, nasal itching, nasal congestion, and sneezing (4). It is not a life-threatening disease, but has a negative impact on patients’ social relationships, reduces patients’ self-confidence, disturbs sleep, causes disorder in daily life performance, and overall reduces patients’ quality of life (5). 

Several immune cells and cytokines have a role in AR pathogenesis. Imbalance in T CD4^+^ lymphocyte, which can differentiate into T helper type 1 and 2 (TH1 and TH2) plays a pivotal role in the pathogenesis of allergic diseases (6). Unlike healthy people, the shift of TH_1_ to TH_2_ in patients with AR is a well-established immunological phenomenon (7, 8). TH1 produces cytokines such as interferon gamma (IFN-γ) and mediates cellular immunity. Also, IFN-γ has a reciprocal effect on TH2 immune response as this cytokine inhibits TH2 development and also abrogates IgE production by B lymphocyte (9). On the other hand, TH2 lymphocytes by producing cytokines such as interleukin-9 (IL-9), interleukin-4 (IL-4), and interleukin-13 (IL-13) orchestrate humoral immunity. TH2 cells by secreting IL-4 and IL-13 induce B lymphocytes to change antibody class to immunoglobulin E (IgE) isotype. So that, high levels of serum IgE, in allergic individuals indirectly indicate an increase in IL-4 level. Also, IL-4 accompanied by IL-9 recruit inflammatory cells, including eosinophil and mast cells to inflamed tissue (10, 11).

Another subset of T cells is regulatory T (Treg), which by suppressing TH2 and TH1 cells response inhibit the allergic and autoimmune reaction. These CD4^+^ T cells express Forkhead Box P3 (FOXP3), the essential transcription factor for Tregs development, and perform their immunomodulatory effects with several mechanisms such as secretion of IL-10 and Transforming Growth Factor β (TGF-β) as suppressive cytokines (12, 13). According to phenotype and function, Treg cells can be classified in to three distinct subsets: I) naïve Treg (nTreg) with low expression of FOXP3 and CD45RA^+^, which develop in the thymus, II) effector Treg (eTreg) that express FOXP3 highly with CD45RA^-^, III) CD45RA^-^ Treg with low expression of FOXP3 that secrete cytokines such as IL-17, IL-2, or IFN-γ in an inflammatory environment. Recently, investigators demonstrated that IL-1β and IL-6 induce FOXP3 ^low^ CD45RA^-^ Treg to secrete IL-17, whereas TGF-β has an inhibitory effect on this Treg subset development (13). Whereas, nTreg and eTreg have an immunomodulatory effect on the immune system; there is a controversy about the inhibitory function of III Tregs population (FOXP3 low CD45RA^-^) in a previous publication (14, 15).TH17 cells are another subset of CD4+ T lymphocytes that was introduced in 2005. These cells produce inflammatory cytokines such as IL-17A, IL-17F, IL-21, Tumor Necrosis Factor-alpha (TNF-α), and IL-22. IL-17 has a critical role in airway inflammation in patients with AR (7, 16). IL-17 produced mainly by TH17 cells, this inflammatory cytokine recruits neutrophils to the respiratory tract in the patients with AR. The correlation between serum IL-17 levels and severity of allergic symptom in AR patients has been shown in previous studies (17).

Management of AR diseases involves the change in patient’s lifestyle, allergen avoidance, administration of anti-inflammatory drugs, and lastly, allergen-specific immunotherapy (ASIT) (1). ASIT with the aim of achieving a long-term efficacy and symptom improvement has been performed as the best treatment for years. ASIT is divided into build-up and maintenance phases. The build-up phase period in conventional immunotherapy is 3–8 months and the range of build-up phase in cluster immunotherapy is 4–8 weeks; however, the costs and longevity of conventional and cluster immunotherapies are the major drawbacks of these protocols. Rush immunotherapy method (RIT) tried to solve these problems by reducing the build-up phase period to 1–3 days. Immunotherapy has regulatory effects on the immune responses, which can induce tolerance to allergens, but the immunological mechanism of this treatment is not well known (18-20). Gene expression assay is a powerful tool to study and classify human diseases; this method helps investigators in illustrating treatment efficacy and prediction of the side effects (21). Employing this knowledge, this study aimed to evaluate alteration in the expression of the genes FOXP3, TGF-β, IL-17, IFN-γ, IL-10, and IL-4 following ARIT protocol in Iranian patients with allergic rhinitis.

## Materials and Methods

***Study population***

Approval was obtained from the Ethics Committee of Mashhad University of Medical Sciences, Mashhad, Iran, and then this clinical trial study was recorded in Iranian Registry of Clinical Trials (IRCT2017050723235N10). Fifteen patients resident in Mashhad, Iran with AR (age range: 15–55 years, mean age 31.3±10.9 years, 8 women, 7 men) who had a clinical history of AR and positive skin prick test for four common regional aeroallergens (weed mix, tree mix, grass mix , and Salsola), enrolled in this study. Patients who had a history of autoimmune disease, consumption of immunosuppressive drugs due to other allergic diseases, pregnancy, immunocompromised patients, patients who had uncontrolled allergic asthma, and patients who suffered from malnutrition were excluded from the study. All patients gave written informed consent before being included in the study.

***Accelerated rush immunotherapy***

*Premedication*

To reduce the risk of adverse events, three days before Accelerated Rush Immunotherapy all patients received prednisolone 30 mg/12 hr, ranitidine 150 mg/12 hr, montelukast 10 mg/day, and fexofenadine 180 mg/12 hr as premedication.

*Allergen extracts*

ARIT according to patient’s prick test result was performed by using four standard commercial extracts including weed mix (nine patients), grass mix (one patient), tree mix (one patient), and Salsola (four patient), which provided by the Greer Company (www.greerlabs.com).

*Immunotherapy protocol*

All patients underwent ARIT with a three-day schedule, while all of them were hospitalized for safety concern in ARIT period. The details of three days ARIT is outlined in Table 1. After reaching 0.5 ml of 1:1 V/V (maintenance dose) and in the maintenance phase, patients received one injection of maintenance dose, every month.

***Clinical parameters***

We used two standard questionnaires: Sino-Nasal Outcome Test-22(SNOT-2)(22) and Mini Rhinoconjunctivitis Quality of Life (mini-RQLQ) (23) to evaluate patient’s allergy symptoms and quality of life, before and three months after ARIT.

***Sample collection***

In the present study, the 5 cc peripheral blood samples were collected from patients in a tube with Ethylene Diamine Tetra Acetic Acid (EDTA) as an anticoagulant, at the two-time-points: before(0) and three months after intervention (3). The blood samples were centrifuged for 5 min at 2500 RPM, then the total RNA was extracted from the buffy coat layer and cDNA synthesis was performed using commercial kits (www.thermofisher.com) according to manufacturer’s instruction.

***SYBR green real-time PCR***

SYBR Green real-time polymerase chain reaction technique (RT-PCR) was recruited for evaluation of gene expression of cytokines IL-4, IL-17, IL-10, TGF-β, and IFN-γ, and transcription factor FOXP3 before and three months after ARIT. Glyceraldehyde 3-phosphate dehydrogenase (GAPDH) was used as the internal reference gene. The sequence of specific primers is listed in Table 2. Amplification was carried out on Rotor-Gene model Q cycler using Takara Master Mix (#RR820L). The total reaction volume was 10 μl, but target genes were studied at two different cycling condition as follows:

1- Initial denaturation 10 min at 95 ^°^C, 40 cycles consisted of 10 sec at 95 ^°^C, 30 sec at 60 ^°^C, and 20 sec at 72 ^°^C, for TGF-β, IL-10, IL-4, IFN-γ, and GAPDH genes.

2- Initial denaturation 2 min at 120 ^°^C, 50 cycles consisted of 15 sec at 95 ^°^C, 15 sec at 60^ °^C, and 20 sec at 72 ^°^C, for IL-17 and FOXP3 genes.

In order to verify the specificity of our primers and following amplification, analysis of the melting curve (60 to 95 ^°^C) and agarose gel electrophoresis were performed. Also, the amplicon of all genes was sequenced to verify the specificity of primers. At last, the relative gene expressions before and three months after ARIT were calculated using the 2^–(ΔΔCT)^ method (24).

***Statistical analysis***

The SPSS version 16.0 statistic software package was used for statistical analysis. Kolmogorov–Smirnov test was used to compare data distribution at first, then the unpaired student’s t-test was performed to compare the means of relative gene expression change before and after the intervention. A *P*-value less than 0.05 (*P*<0.05) was considered statistically significant. The data were presented as the mean±standard error of the mean (SEM(.

## Results

***Clinical efficacy of ARIT***

**Figure 1:**
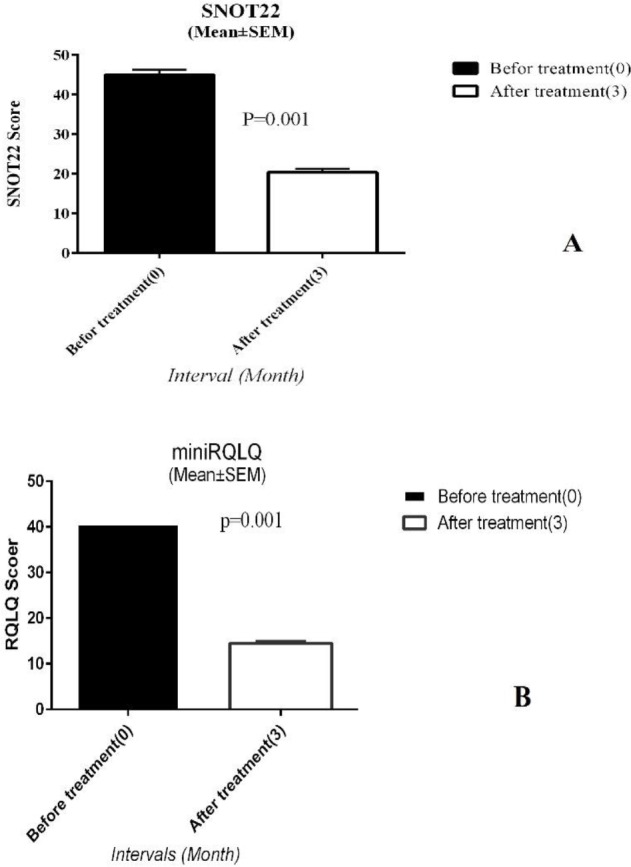
The SNOT-22 (A) and mini-RQLQ (B) questioners score before and three months after ARIT questioners

SNOT: sino-nasal outcome test; RQLQ: Rhinoconjunctivitis Quality of Life

The mean of SNOT-22 questioner score in 15 patients before ARIT was 45.7±4.9, and three months after ARIT significantly decreased to 20.7±3.3 (*P*-value<0.001)**, **indicating an improvement of Sino-Nasal allergic symptoms (Figure 1-A). The mean of mini-RQLQ questioner score at the baseline was 38.13±4.90 and three months after ARIT significantly decreased to 13.63±1.83 (*P*-value<0.001), reduction in mini-RQLQ questioner score after treatment indicates an improvement of patients’ daily performance and quality of life (Figure 1-B).

***Relative gene expression***

*Gene expression of FOXP3, IL-10, and TGF-β before and after ARIT*

**Figure 2:**
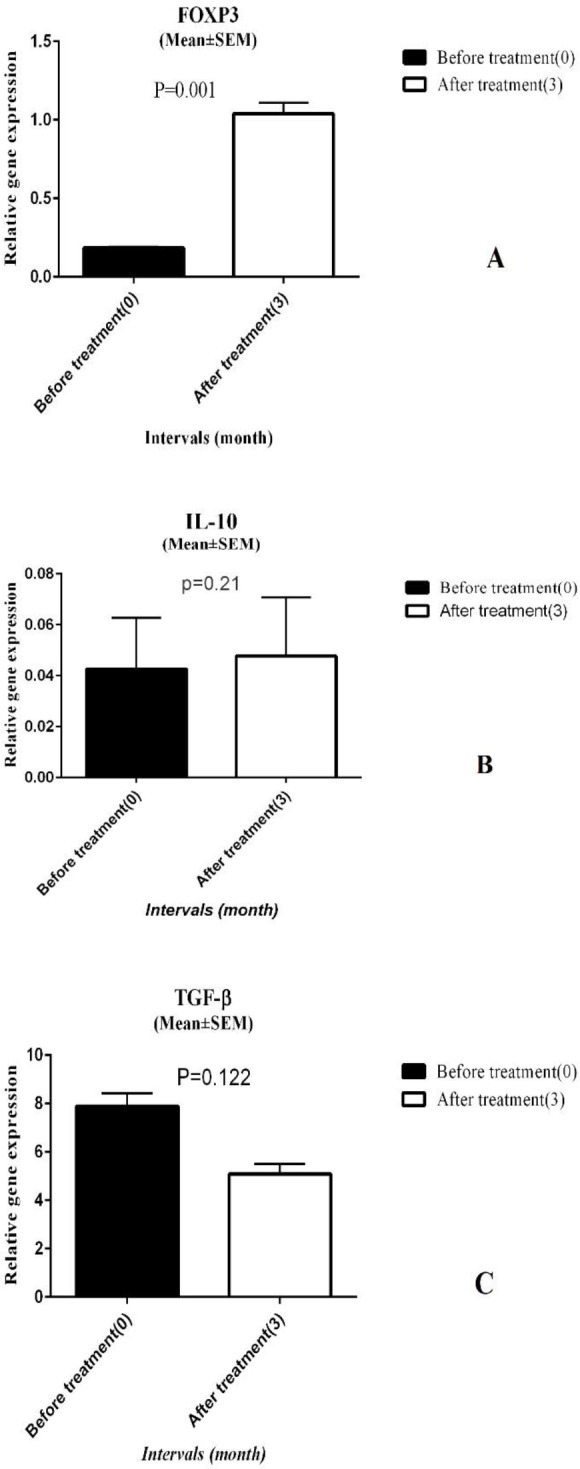
The relative expression levels of FOXP3 (A), IL-10 (B), and TGF-β (C) genes at baseline and three months after ARIT

This study considered FOXP3, IL-10, and TGF-β genes as Tregs representatives. Three months after ARIT, FOXP3 and IL-10 gene expression increased 4.3 fold and 1.01 fold, respectively, but only FOXP3 gene alteration was statistically significant (*P*-value<0.001) (Figures 2A and 2B). On the other hand, TGF-β gene expression declined 0.24 fold, compared with baseline, but this decline was not significant (*P*-value>0.05) (Figure 2C).

*IFN-γ relative gene expression*

**Figure 3:**
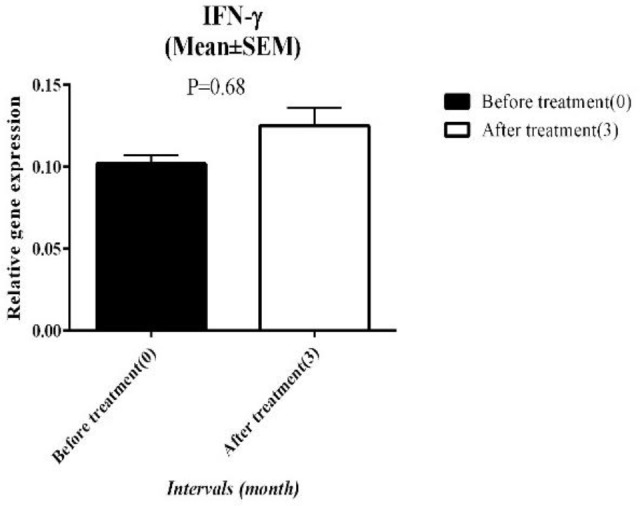
Relative IFN-γ expression before and after ARIT

To assess the ARIT effect on TH1 cells, we used the relative expression of IFN-γ as a key cytokine of these cells. Before the intervention, the mean of IFN-γ gene expression was 0.10±0.02 (mean±SEM) and three months after ARIT it increased to 0.12±0.04 and overexpressed 1.2 fold compared to baseline, although this alteration was not significant (*P*-value>0.05). (Figure 3).

*IL-4 relative gene expression*

**Figure 4:**
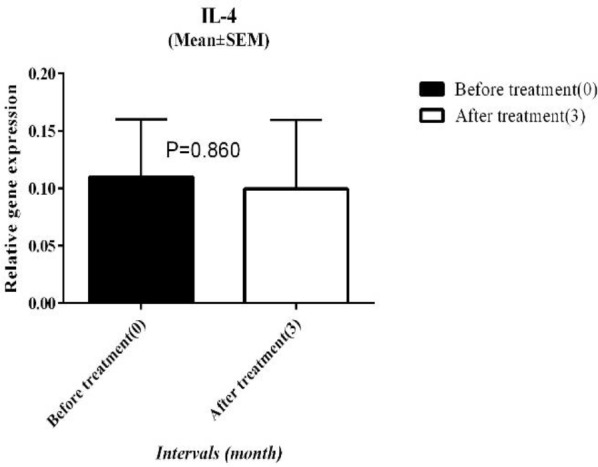
The relative gene expression of IL-4 at the baseline and 3 months after ARIT

Before ARIT the mean expression level of IL-4 was 0.11±0.05 and 3 months after ARIT it changed to 0.09±0.06, but this down-regulation was not significant (*P*-value>0.05)(Figure 4).

*IL-17 relative gene expression*

**Figure 5:**
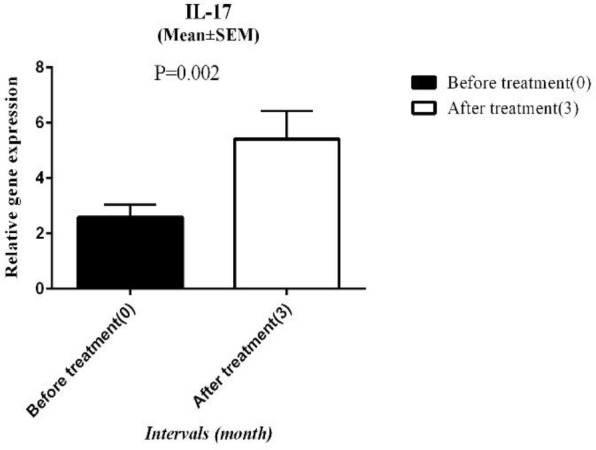
Relative IL-17 gene expression at the baseline and 3 months after ARIT

In the present study, IL-17 as an inflammatory cytokine was considered a signature cytokine of the TH17 cell. The relative expression of IL-17 was upregulated to 2.7 fold change compared to baseline. The mean expression of IL-17 before ARIT was 2.58±0.46 and this value significantly increased to 5.41±1.02 at the end of the study (Figure 5).

## Discussion

The alteration of competent immune cell gene expression in the peripheral blood cells of Iranian patients with AR who underwent ARIT was studied. Immunotherapy was performed with modified protocol by the allergist in the allergy ward of Ghaem Hospital, in Mashhad University of Medical Sciences, Iran. Three months after ARIT, our results demonstrated that this treatment, significantly decreases the allergic symptoms and improves quality of life in patients with AR (*P*>0.05), also our data indicated that the expression of FOXP3 and IL-17 genes had a significant rise in patient’s peripheral blood samples, three months after ARIT compared to baseline. We observed down-regulation of TGF-β and IL-4 genes and up-regulation of IFN-γ in the peripheral blood samples, although these alterations were not statistically significant. 

ARIT: Accelerated Rush Immunotherapy; TGF-β: Transforming Growth Factor β

ARIT: Accelerated Rush Immunotherapy

ARIT: Accelerated Rush Immunotherapy

ARIT: Accelerated Rush Immunotherapy

A Japanese scholar, Sakaguch, discovered CD4^+^CD25^+^Treg cells in 1995 and explained the role of these cell on body’s immune homeostasis, nowadays 22 subsets of human Treg cells have been described according to the expression of different surface markers (15, 25). FOXP3 as a specific transcription factor of Tregs lineage was employed for monitoring nTreg (Natural regulatory T cell) during immunotherapy in many previous studies (6, 26, 27). Derivation from thymus and high expression of FOXP3, are two specific characterizes of nTregs. In the present study, we measured the impact of ARIT on Treg by evaluating the FOXP3 expression at three months following ARIT. In addition to the clinical improvement, we observed up-regulation of FOXP3 gene expression (4.3 fold) three months after ARIT. This finding resembled other studies that reported a significant rising in FOXP3 expression in the patient with AR one year after subcutaneous immunotherapy (SIT)(25). Urra *et al*. reported insignificant increasing in FOXP3 expression six months and one year after IT in the patient with AR (27). These data showed that the increase in peripheral Tregs and/or their function following ARIT might be related to modulation of the immune response pathway and improving clinical symptoms of patients.

**Table 1:** Accelerated rush immunotherapy schedule

First day		
Concentration (v/v)	Dose (ml)	Interval (min)
1:10،000	0.05	00:00
1:10،000	0.3	00:15
1:1،000	0.1	00:30
1:1،000	0.3	00:30
1:100	0.1	60:00
1:100	0.3	60:00
1:10	0.1	60:00
1:10	0.2	60:00
Second day		
Concentration (v/v)	Dose (ml)	Interval (min)
1:10	0.2	00:00
1:10	0.4	60:00
1:1	0.05	60:00
Third day		
Concentration (v/v)	Dose (ml)	Interval (min)
1:1	0.1	00:00
1:1	0.3	60:00
1:1	0.5	60:00

**Table 2:** Specific primers sequences to evaluate the gene expression for IFN-γ, IL-4, IL-10, IL-17, TGF-β, and FOXP3

** Sequence **	**Primer**	**Product Length**	**Accession Number**	**Name**
5’-CACTAGGCGCTCACTGTTCTC-3’	Forward	101 bp	NM_001289746.1	GAPDH
5’-CCAATACGACCAAATCCGTTGAC-3’	Reverse			
5’-GAGTGTGGAGACCATCAAGGAAG-3’	Forward	124 bp	NM_000619	IFN-γ
5’-TGCTTTGCGTTGGACATTCAAGTC-3’	Reverse			
5’-CCGTAACAGACATCTTTGCTGCC-3’	Forward	108 bp	NM_000589	IL-4
5’-GAGTGTCCTTCTCATGGTGGCT-3’	Reverse			
5’-TCTCCGAGATGCCTTCAGCAGA-3’	Forward	126 bp	NM_000572	IL-10
5’-TCAGACAAGGCTTGGCAACCC A-3’	Reverse			
5’-CGGACTGTGATGGTCAACCTGA-3’	Forward	156 bp	NM_002190	IL-17
5’-GCACTTTGCCTCCCAGATCACA-3’	Reverse			
5’-TACCTGAACCCGTGTTGCTCTC-3’	Forward	122 bp	NM_000660	TGF-β
5’-GTTGCTGAGGTATCGCCAGGAA-3’	Reverse			
5’-GGCACAATGTCTCCTCCAGAGA-3’	Forward	128 bp	NM_001114377	FOXP3
5’-CAGATGAAGCCTTGGTCAGTGC-3’	Reverse			

According to earlier studies, Tr_1 _is an inducible, regulatory FOXP3^-^ T cell that has an inhibitory effect by secreting IL-10 and TGF-β cytokines predominantly (28, 29). Tr_1 _cells based on the expression of inducible T-cell costimulator (ICOS) have been categorized in two different subsets: I) iTreg ICOS^+^, which predominantly secret IL-10 and II) and iTreg ICOS^- ^, which perform their suppressive role by producing TGF-β (18). The decline of Tr_1 _cells in the allergic patient compared to healthy individuals was found in a previous study (30). Our study, by evaluating gene expression of IL-10 and TGF- β, tried to investigate the impact of ARIT on T reg.

IL-10 shows inhibitory effect on both TH1 and TH2 cells (31). This study showed increases in the expression of IL-10 three months after ARIT although this up-regulation was not significant the result was in concordance with results of previous studies (27) that reported a significant increase in IL-10-producing CD4^+ ^T cells, six and twelve months after IT(27). An Iranian study also reported a significant increase in serum IL-10 levels, in AR patients, six months after SLIT (32). Given that, increased ICOS^+^, IL-10-secreting Tr_1_ in peripheral blood of AR patients, may be one of the immunological mechanisms of ARIT. However, simultaneous monitoring of these cells by two specific markers of Tr_1_ cells, including ICOS and IL-10, employing flow cytometry is suggested for future studies.

Alteration in TGF-β in patients, which underwent IT, is controversial. For example, liu *et al*. reported two-fold overexpression of TGF-β in a patient with AR three months after IT. However, this rising was not significant (18). While another study recorded significant increase in TGF-β gene expression, six months after sublingual immunotherapy (SLIT), in a patient with AR (33). Contrary to the two above mentioned studies, we found studies that reported no change in TGF-β secretion levels in AR patient PBMCs 1.5–2 years after IT (12). The decline in TGF-β gene expression in our patients was not in concordance with our hypothesis, but the assessment of TGF-β gene expression by its own, in whole blood cells may be not a good idea to monitor ICOS^- ^Tr_1 _function during ARIT_.  _On other hand, TGF-β produced by other immune cells, such as TH2 and so decline in this cytokine gene expression might be related to a decrease in number or function of TH2 cells.IL-17, as an inflammatory cytokine predominantly is produced by TH17, and it is responsible for the recruitment of neutrophils and eosinophils to inflamed tissue (34). According to previous studies, IL-17 not only has a positive correlation with the severity of allergic symptoms, but also it can be used as a biomarker for monitoring the efficacy of IT (35, 36). In the present study, we examined the IL-17 expression before and three months after the intervention, for evaluating the impact of ARIT on TH17 cells. Surprisingly, and despite significant clinical improvement three months after ARIT, IL-17 gene expression was up-regulated markedly as 2.7 fold-change compared to baseline. This finding was contrary to earlier studies (36, 37) that reported decreased TH17 count and IL-17 serum levels after specific immunotherapy (SIT). 

In addition to TH17 other immune cells such as memory TCD_8_^+^, Tγδ, natural killer (NK), and macrophages also secret IL-17(38). So increased IL-17 is not necessarily representative of TH17 function. Also, a subtype of FOXP3^+ ^Tregs in inflammatory condition can produce IL-17. These regulatory T cells simultaneously express Retinoic acid-related Orphan Receptors (RORγT), a specific transcription factor of TH17 lineage, and chemokine receptor 6 (CCR6)(17). New literature emphasizes that IL-17 secretor Treg cells have human leukocyte antigen DR^-^(HLA-DR^-^) and in proinflammatory condition (in presence of IL-1β and IL-6) can secret IL-17. These TH17 Tregs are also blocked with TGF-β (13). 

In the present study, down-regulation of TGF-β and overexpression of FOXP3 was observed three months after ARIT, which might be facilitated condition shifting towards HLA-DR^-^ Treg subtype. Employing this knowledge, the significant increase in the IL-17 gene expression following ARIT might be related to the HLA-DR^-^ FOXP3+ Tregs subtype. Also, we evaluated the IL-17 expression, three months after ARIT, whereas previous studies measured IL-17 at one or two years after IT. It would be more likely that IL-17 is raised at the initiation phase of ARIT and declines over time. To get a better conclusion, we suggest measurement of serum IL-17 and RORγT as specific markers of TH17 cells in addition to IL-17 gene expression in a longer follow up period.

 IFN-γ is a well-known inflammatory cytokine that secrets from TH1, and in many studies was employed to evaluate TH1 activity during IT. In this study, we evaluated TH1 response following ARIT, by evaluating IFN-γ gene expression before and three months after ARIT. Although in our study gene expression of IFN-γ compared to baseline was increased (1.2 fold) following ARIT, but this up-regulation was not significant. Findings in the present study were similar to some previous studies that reported no significant increase in serum levels of IFN-γ, fifteen months after IT (9), while other studies have exhibited a significant increase in IFN-γ secretor T cells, three months after SLIT (39). A possible reason that we could not determine statistical significance in our result may be related to the small size of samples or differentiation in IT protocols. Taken together, we observed a shift towards the production of IFN-γ, whereas IL-4 as hallmark cytokine for TH2 was decreased. These data indicate a shift from TH2 to TH1 cells in the early phase of ARIT. However, the small sample size or short period of ARIT follow up are probably reasons that these results could not achieve statistical significance.

Previous investigations reported an increase in IL-4 secreting T cells in peripheral blood of allergic patients (28). IL-4, IL-5, and IL-13 are produced by TH2 type cells and have cardinal roles in allergic rhinitis. TH2 cells by secreting IL-4 improve TH2 self-development and also induce B lymphocytes to synthesis specific IgE against allergens (9, 12). This study aimed to investigate the changes in TH2 cells, by assessment of IL-4 gene expression alteration in the peripheral blood of patients with AR, before and three months after ARIT. We observed IL-4 expression after ARIT decreased 0.76 fold compared to baseline, but this decline did not reach statistical significance. Results of the present study are similar to the findings of some earlier studies (37) that demonstrated a significant decrease in IL-4 levels three years after SLIT. Wei *et al*. (12) also addressed a decline in IL-4 production by TH2 cells after SIT in children with allergic asthma who underwent immunotherapy for 1.5–2 year periods. Together, these data indicate that IT in standard or accelerated protocols, might suppress IL-4 expression and cause alleviation of allergic symptoms. Some limitations of the present study need to be considered: small pilot study sample size, financial constraints for simultaneous assay of cell phenotypes, especially RORγt and HLA-DR by the flow cytometry technique, also measurement of the serum levels of target cytokines.

## Conclusion

A significant increase in the gene expression of FOXP3 and IL-17 in parallel with remarkable clinical improvement following the ARIT protocol might be related to increases in HLA-DR^-^ FOXP3+ regulatory subtype (IL-17 producer) at the initiation phase of ARIT. Employing the flow cytometry technique to evaluate the phenotype of regulatory T-cell subsets (especially HLA-DR) as well as analysis of expression of FOXP3, RORγT, and IL-17 genes with longer follow-up periods might be a good choice for future studies. 

## References

[1] Kakli HA, Riley TD (2016). Allergic rhinitis. Prim Care.

[2] Varshney J, Varshney H (2015). Allergic rhinitis: an overview. Indian J Otolaryngol Head Neck Surg.

[3] Greiner AN, Hellings PW, Rotiroti G, Scadding GK (2012). Allergic rhinitis. Lancet.

[4] Min Y-G (2010). The pathophysiology, diagnosis and treatment of allergic rhinitis. Allergy Asthma Immunol Res.

[5] Bousquet J, Khaltaev N, Cruz AA, Denburg J, Fokkens W, Togias A (2008). Allergic rhinitis and its impact on asthma (ARIA) 2008. Allergy.

[6] Lee S-M, Gao B, Dahl M, Calhoun K, Fang D (2009). Decreased FoxP3 gene expression in the nasal secretions from patients with allergic rhinitis. Otolaryngol Head Neck Surg.

[7] Bayrak Degirmenci P, Aksun S, Altin Z, Bilgir F, Arslan I, Colak H (2018). Allergic Rhinitis and Its Relationship with IL-10, IL-17, TGF-β, IFN-γ, IL 22, and IL-35. Dis Markers.

[8] Baumann R, Rabaszowski M, Stenin I, Tilgner L, Scheckenbach K, Wiltfang J (2013). Comparison of the nasal release of IL-4, IL-10, IL-17, CCL13/MCP-4, and CCL26/eotaxin-3 in allergic rhinitis during season and after allergen challenge. Am J Rhinol Allergy.

[9] Boghdadi G, Marei A, Ali A, Lotfy G, Abdulfattah M, Sorour S (2012). Immunological markers in allergic rhinitis patients treated with date palm immunotherapy. Inflammation Res.

[10] Maggi E, Vultaggio A, Matucci A (2012). T-cell responses during allergen-specific immunotherapy. Curr Opin Allergy Clin Immunol.

[11] Micheal S, Minhas K, Ishaque M, Ahmed F, Ahmed A (2013). IL4 gene polymorphisms and their association with atopic asthma and allergic rhinitis in Pakistani patients. J Investig Allergol Clin Immunol.

[12] Wei W, Liu Y, Wang Y, Zhao Y, He J, Li X (2010). Induction of CD4+ CD25+ Foxp3+ IL-10+ T cells in HDM-allergic asthmatic children with or without SIT. Int Arch Allergy Immunol.

[13] Beriou G, Costantino CM, Ashley CW, Yang L, Kuchroo VK, Baecher-Allan C (2009). IL-17–producing human peripheral regulatory T cells retain suppressive function. Blood.

[14] Afzali B, Mitchell PJ, Edozie FC, Povoleri GA, Dowson SE, Demandt L (2013). CD161 expression characterizes a subpopulation of human regulatory T cells that produces IL-17 in a STAT3-dependent manner. Eur J Immunol.

[15] Mohr A, Malhotra R, Mayer G, Gorochov G, Miyara M (2018). Human FOXP 3+ T regulatory cell heterogeneity. Clin Transl Immunology.

[16] Llosa NJ, Geis AL, Orberg ET, Housseau F (2014). Interleukin-17 and type 17 helper T cells in cancer management and research. Immunotargets Ther.

[17] Nieminen K, Valovirta E, Savolainen J (2010). Clinical outcome and IL-17, IL-23, IL-27 and FOXP3 expression in peripheral blood mononuclear cells of pollen-allergic children during sublingual immunotherapy. Pediatr Allergy Immunol.

[18] Liu Z, Yelverton R, Kraft B, Tanner S, Olsen N, Aune T (2005). Highly conserved gene expression profiles in humans with allergic rhinitis altered by immunotherapy. Clin Exp Allergy.

[19] Ring J, Gutermuth J (2011). 100 years of hyposensitization. history of allergen-specific immunotherapy (ASIT). Allergy.

[20] Calabria CW (2013). Accelerated immunotherapy schedules. Curr Allergy Asthma Rep.

[21] Davis LS, Bhutani S, Barnett SR, Khan DA (2011). Early gene expression changes with rush immunotherapy. Clin Mol Allergy.

[22] Lachanas VA, Tsea M, Tsiouvaka S, Hajiioannou JK, Skoulakis CE, Bizakis JG (2014). The sino-nasal outcome test (SNOT)-22: validation for Greek patients. Eur Arch Otorhinolaryngol.

[23] Van Oene C, Van Reij E, Sprangers M, Fokkens W (2007). Quality-assessment of disease-specific quality of life questionnaires for rhinitis and rhinosinusitis a systematic review. Allergy.

[24] Giulietti A, Overbergh L, Valckx D, Decallonne B, Bouillon R, Mathieu C (2001). An overview of real-time quantitative PCR: applications to quantify cytokine gene expression. Methods.

[25] Zheng R, Wu X, Huang X, Chen Y, Yang Q, Li Y (2014). Gene expression pattern of Treg and TCR Vγ subfamily T cells before and after specific immunotherapy in allergic rhinitis. J Transl Med.

[26] Xu G, Mou Z, Jiang H, Cheng L, Shi J, Xu R (2007). A possible role of CD4+ CD25+ T cells as well as transcription factor Foxp3 in the dysregulation of allergic rhinitis. Laryngoscope.

[27] Urra J, Carrasco P, Feo-Brito F, De La Roca F, Guerra F, Cabrera C (2014). Immunotherapy reduces CD40L expression and modifies cytokine production in the CD4 cells of pollen allergy patients. J Investig Allergol Clin Immunol.

[28] Han D, Wang C, Lou W, Gu Y, Wang Y, Zhang L (2010). Allergen-specific IL-10-secreting type IT regulatory cells, but not CD4+ CD25+ Foxp3+ T cells, are decreased in peripheral blood of patients with persistent allergic rhinitis. Clin Immunol.

[29] Sabat R, Grütz G, Warszawska K, Kirsch S, Witte E, Wolk K (2010). Biology of interleukin-10. Cytokine Growth Factor Rev.

[30] Lou W, Wang C, Wang Y, Han D, Zhang L (2012). Responses of CD4+ CD25+ Foxp3+ and IL-10-secreting type IT regulatory cells to cluster-specific immunotherapy for allergic rhinitis in children. Pediatr Allergy Immunol.

[31] Savolainen J, Laaksonen K, Rantio-Lehtimäki A, Terho E (2004). Increased expression of allergen-induced in vitro interleukin-10 and interleukin-18 mRNA in peripheral blood mononuclear cells of allergic rhinitis patients after specific immunotherapy. Clin Exp Allergy.

[32] Ahmadiafshar A, Taymourzadeh B, Shaikhi A, Mazloomzadeh S, Torabi Z (2013). Evaluation of IL10, TGF-B and Specific IgE and IgG Levels during Sublingual Rye Grass Immunotherapy. J Aller Ther.

[33] Hoseini R, Jabbari F, Rezaee A, Rafatpanah H, Yousefzadeh H, Ariaee N (2018). House dust mite sublingual-swallow immunotherapy in perennial rhinitis: a double-blind, placebo-controlled Iranian study. J Biol Regul Homeost Agents.

[34] Krishna M, Huissoon A (2011). Clinical immunology review series: an approach to desensitization. Clin Exp Immunol.

[35] Pawankar R, Hayashi M, Yamanishi S, Igarashi T (2015). The paradigm of cytokine networks in allergic airway inflammation. Curr Opin Allergy Clin Immunol.

[36] Li CW, Lu HG, Chen DH, Lin ZB, Wang DY, Li TY (2014). In vivo and in vitro studies of Th17 response to specific immunotherapy in house dust mite-induced allergic rhinitis patients. PLoS One.

[37] Li H, Xu E, He M (2015). Cytokine responses to specific immunotherapy in house dust mite-induced allergic rhinitis patients. Inflammation.

[38] Jin W, Dong C (2013). IL-17 cytokines in immunity and inflammation. Emerg Microbes Infect.

[39] Ciprandi G, Fenoglio D, Di Gioacchino M, Ferrera A, Ferrera F, Sormani M (2008). Sublingual immunotherapy provides an early increase of interferon-gamma production. Biol Regul Homeost Agents.

